# Development and Pilot Validation of the Sexual Satisfaction and Emotional Impact After Cesarean Section Scale (SSEI-CS-24): A Pilot Study

**DOI:** 10.7759/cureus.100253

**Published:** 2025-12-28

**Authors:** Ana-Maria Brezeanu, Dragos Brezeanu, Stase Simona, Dan Cozmei, Vlad I Tica

**Affiliations:** 1 6th Department, Faculty of Medicine, Ovidius University, Constanta, ROU

**Keywords:** body image, cesarean section, interpersonal relationships, postpartum depression, scale validation, sexual satisfaction

## Abstract

Background

Cesarean section (CS) scars may negatively affect sexual function, body image, and emotional well-being, yet no validated tool specifically addresses these outcomes.

Objective

To develop and pilot-validate the Sexual Satisfaction and Emotional Impact after Cesarean Section Scale (SSEI-CS-24), a 24-item instrument assessing sexual, psychological, and relational adaptation after CS.

Methods

The SSEI-CS-24 was developed through literature review, qualitative interviews, and expert consensus. Content validity was quantified with the Content Validity Index. Fifty women completed the scale at 40 days postpartum. Reliability was assessed with Cronbach’s α and intraclass correlation coefficient (ICC), while construct validity was examined using exploratory factor analysis (principal component analysis with Varimax rotation).

Results

The SSEI-CS-24 demonstrated excellent internal consistency (Cronbach’s α=0.89) and good test-retest reliability (ICC=0.84). Factor analysis supported a five-domain structure - sexual satisfaction, body image/self-esteem, anxiety and stress, interpersonal relationships, and support/resources - explaining 68% of variance. Distributions were normal without floor or ceiling effects.

Conclusions

The SSEI-CS-24 demonstrates strong preliminary psychometric properties and shows promise as a multidimensional tool for assessing the sexual and emotional impact of cesarean section scars. Further confirmatory validation in larger, multicenter cohorts is warranted before routine clinical use.

## Introduction

Cesarean section (CS) delivery has become increasingly common across diverse healthcare systems, yet its psychosocial consequences remain poorly characterized [1]. Beyond the immediate postoperative period, the presence of a surgical scar may influence how women perceive their bodies, relate emotionally to childbirth, and experience intimacy during the postpartum phase [2]. Although previous research suggests that negative body image and changes in sexual functioning may emerge after CS, current evidence is fragmented and does not clearly integrate these dimensions into a unified framework [3].

Postpartum emotional vulnerability, including anxiety, reduced self-esteem, and altered partner communication, has been associated with both physical recovery and subjective perceptions of the birth experience [4,5]. Existing assessment instruments typically focus either on sexual function or on mood disturbances, leaving a methodological gap in evaluating the combined sexual-emotional impact of cesarean scars. Although instruments such as the Female Sexual Function Index (FSFI) and the Edinburgh Postnatal Depression Scale (EPDS) are widely used, they capture only partial aspects of postpartum outcomes, focusing either on sexual function or mood disorders [5-7]. Predictors of postpartum body dissatisfaction include maternal body mass index (BMI), mode of delivery, and sociocultural pressures [8]. The type of birth may also affect sexual outcomes through both physiological and psychological mechanisms. Perineal trauma, for example, can directly impair sexual function by causing pain, scarring, and pelvic floor dysfunction, while also contributing indirectly through psychological pathways such as body image concerns and anxiety. Breastfeeding difficulties and negative birth experiences may further exacerbate these effects [9,10]. These physical and psychological changes significantly influence self-esteem and body appreciation, while insufficient social support increases the risk of anxiety and depressive symptoms [11,12].

Currently, there is no validated instrument to comprehensively assess the combined sexual, emotional, and relational impact of scar-related impact on women’s postpartum well-being [13].

To address this gap, we developed the Sexual Satisfaction and Emotional Impact after Cesarean Section Scale (SSEI-CS-24), a 24-item self-report instrument designed to evaluate five domains: sexual satisfaction, body image and self-esteem, anxiety and stress, interpersonal relationships and intimacy, and support and resources. This study describes the development process of the SSEI-CS-24 and presents the results of its pilot validation in a cohort of postpartum women.

## Materials and methods

Study design and participants

We carried out a prospective pilot investigation including 50 women who had undergone cesarean delivery and returned for routine evaluation approximately 40 days after birth. Participants were recruited consecutively from a tertiary obstetric unit serving an urban-rural mixed population.

The study protocol received approval from the institutional ethics committee (approval no. SCJU 16839/15.12.2021), and all participants signed informed consent permitting both participation and anonymous data use.

Eligibility criteria included women aged 18-40 years, with singleton pregnancies and live neonates delivered by CS. Exclusion criteria were major obstetric complications (postpartum hemorrhage, severe infection), non-standard methods of analgesia, other sources of psychological stress due to obstetrical conditions or history, as we described a documented history of psychiatric disorders, or refusal to participate [14].

Demographic and clinical data were extracted from medical records and included maternal age, parity, body mass index (BMI), perioperative hematological parameters (hemoglobin, hematocrit, leukocytes, platelets, pre- and postoperatively), and indicators of postoperative recovery.

The sample size for this study was pragmatically set at 50 participants, in line with recent recommendations for pilot psychometric validation, where smaller samples are acceptable to explore preliminary reliability and construct validity before confirmatory testing [13]. Although the conventional rule of three to five participants per item would suggest a larger sample, our design was explicitly exploratory and aimed to provide pilot evidence to guide subsequent multicenter validation studies.

Instrument development: the Sexual Satisfaction and Emotional Impact after Cesarean Section Scale

The Sexual Satisfaction and Emotional Impact after Cesarean Section Scale (SSEI-CS-24) was developed in three stages. First, items were created based on an extensive literature review, qualitative interviews with postpartum women (n=10), and an expert consensus panel including gynecologists, psychologists, and midwives. 

To ensure methodological rigor and strengthen content validity, the expert panel was composed of seven professionals purposefully selected for their complementary expertise in postpartum care and psychometric evaluation. Eligibility was based on established professional experience in obstetrics and gynecology, clinical psychology, midwifery, or nursing, with all members having at least five years of clinical or academic practice. Selection further emphasized direct involvement in maternal health, sexual function, and psychological adaptation after childbirth, as well as scholarly contributions such as peer-reviewed publications and teaching activity in related fields. Fluency in Romanian was required for evaluating the original item pool, while sufficient knowledge of English was considered essential for the cross-linguistic adaptation of the instrument. The final panel consisted of three consultant obstetrician-gynecologists with expertise in cesarean delivery and maternal morbidity, two clinical psychologists specializing in perinatal mental health, one senior midwife with extensive intrapartum and postpartum care experience, and one nursing academic with a research focus on women’s health outcomes.

Second, experts evaluated each item for clarity, relevance, and representativeness. The Content Validity Index (CVI) was calculated at both the item level (I-CVI) and the scale level (S-CVI), with thresholds ≥0.80 considered acceptable [15].

The SSEI-CS-24 was then pilot-tested by administering it to 50 women at 40 days postpartum. The instrument was originally developed and validated in Romanian, the participants’ native language (Appendix B). For dissemination purposes, an English translation was produced by a professional medical translator, reviewed by the research team, and finalized by consensus (Appendix A). Importantly, only the Romanian version has been pilot-validated for use; the English version is provided for reference and has not yet undergone psychometric validation. Cross-cultural adaptation and validation will be required before international use.

Items were rated on a five-point Likert scale (1=strongly disagree to 5=strongly agree). Negatively worded items were reverse-coded so that higher scores consistently reflected greater sexual satisfaction and better psychological adaptation. Domain scores were calculated by summing relevant items, while the total score was the sum of all items (range 24-120). Total score was calculated as the sum of all items (range: 24-120), with higher scores indicating greater sexual satisfaction, better self-esteem, lower anxiety/stress, stronger interpersonal relationships, and better perceived support.

The final version comprised 24 items, grouped into five domains as follows:

1. Sexual satisfaction (items 1-5)

2. Body image and self-esteem (items 6-10)

3. Anxiety and stress (items 11-15)

4. Interpersonal relationships and intimacy (items 16-20)

5. Support and resources (items 21-24).

The assessment was scheduled at 40 days postpartum, corresponding to the conventional six-week follow-up visit, which marks the end of the puerperium and is a standard time point for postpartum evaluation in Romania and many European settings. We acknowledge, however, that most women resume sexual activity between six and eight weeks, and that our assessment at 40 days primarily reflects early perceptions of scar-related discomfort, body image, and emotional adaptation, rather than full sexual activity resumption. This time frame is widely adopted in clinical practice and supported by international guidelines, as the postpartum period is typically defined as lasting six to eight weeks after childbirth. The World Health Organization, as well as European and UK guidelines, recommend structured postnatal care during this interval, making the 40-day evaluation both clinically relevant and evidence-based [16,17].

Translation and Cross-cultural Adaptation

The English version of the SSEI-CS-24 was developed following established guidelines for cross-cultural adaptation of patient-reported outcome measures. The translation process consisted of three sequential steps. First, forward translation was independently performed by two bilingual translators working separately, whose native language was English and who were fluent in Romanian. Second, a backward translation was conducted independently by two different bilingual translators, blinded to the original version, whose native language was Romanian. Third, all versions were reviewed by a multidisciplinary expert committee, including obstetrician-gynecologists, psychologists, and researchers experienced in scale development, who evaluated semantic, idiomatic, conceptual, and experiential equivalence. Discrepancies were resolved by consensus, and the final English version was approved for reference use.

Importantly, only the Romanian version underwent psychometric testing in this pilot study. The English version has not yet been formally validated and will require independent psychometric evaluation in future studies prior to clinical or research use.

Statistical analysis

Descriptive analyses were performed for demographic characteristics and scale responses. Internal consistency was examined using Cronbach’s α for total and subscale scores. Temporal stability was tested in a subgroup of 20 participants using intraclass correlation coefficients based on a two-way mixed model. Exploratory factor analysis was performed using principal component analysis (PCA) with Varimax rotation. PCA was selected due to the exploratory nature of this pilot study and the limited sample size, as it provides a robust and parsimonious approach for identifying underlying dimensional structures and reducing data complexity in early-stage instrument development. At this stage, the primary aim was to explore the potential factor structure and item clustering rather than to model latent constructs. Confirmatory factor analysis (CFA) was not performed, as the current sample size was insufficient; CFA is planned in future studies with larger, independent cohorts to formally test the factorial validity of the SSEI-CS-24.

## Results

General characteristics of the sample

**Figure 1:**
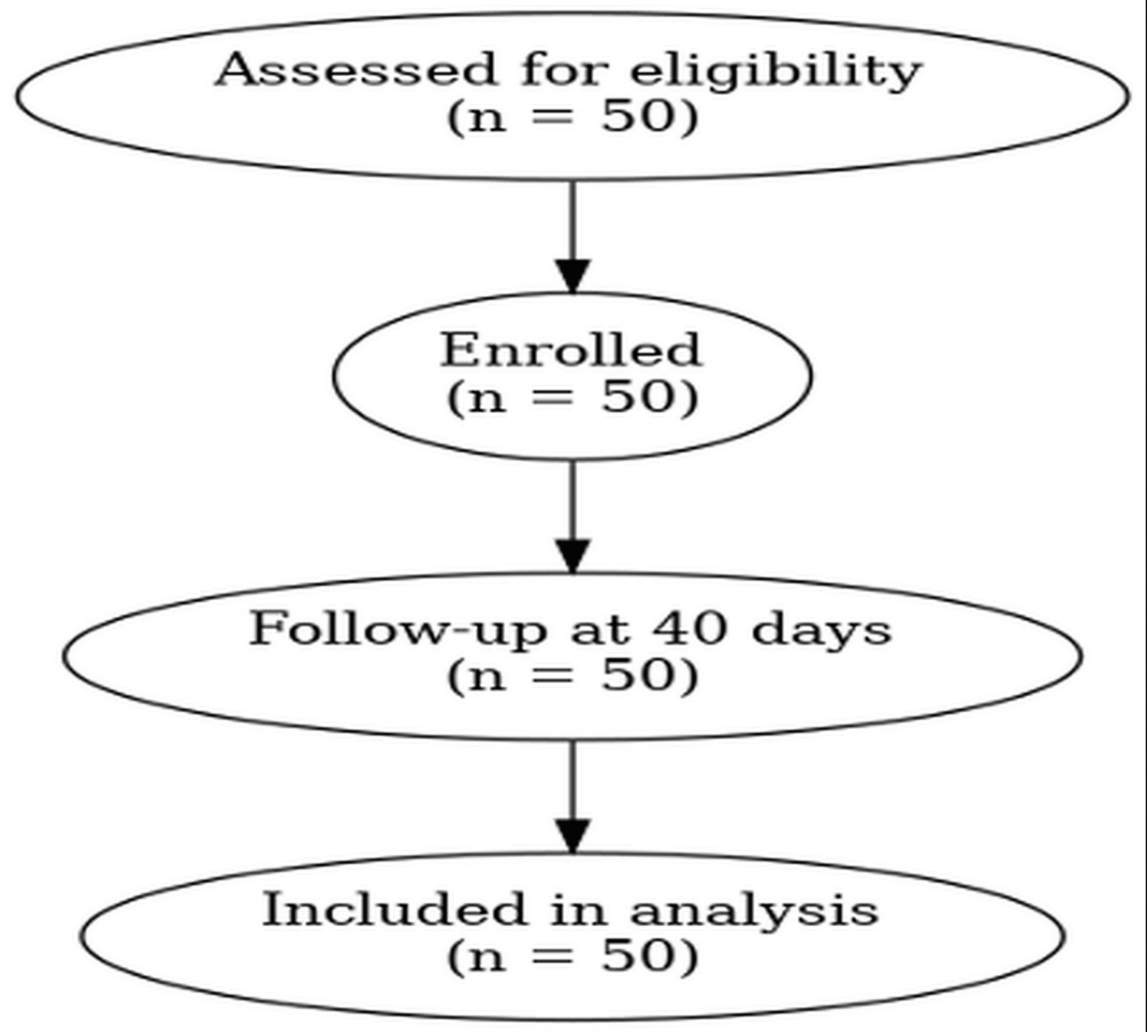
Participant flow diagram A total of 50 eligible women who delivered by cesarean section were consecutively recruited and assessed at the 40-day postpartum visit. No participants were lost to follow-up, and there were no exclusions after enrollment, resulting in a final analytic sample of 50 women with complete questionnaire data.

SSEI-CS-24 was administered to a cohort of 50 women at 40 days postpartum following cesarean section. The mean age was 27.9±6.1 years (range 18-40 years), with a balanced distribution between primiparous (52.0%) and multiparous women (48.0%). The response rate was 100%, with no missing data reported (Figure 1 and Table 1).

**Table 1:** Patient Demographics The study cohort consisted of 50 postpartum women who delivered by cesarean section. The mean maternal age was 27.9±6.1 years (range 18–40 years), reflecting a typical reproductive-age population. Parity was nearly balanced, with 52% primiparous and 48% multiparous participants.

Characteristic	Value
Age (years)	27.9±6.1 (range 18–40)
Parity (number of women (%))	Primiparous: 26 (52.0%)
	Multiparous: 24 (48.0%)
Delivery mode (number of women (%))	Cesarean: 50 (100%)

Internal consistency and reliability

The scale demonstrated high internal consistency, with a Cronbach’s α of 0.89 for the total score. Subscale α values ranged between 0.78 and 0.86, exceeding the conventional threshold of 0.70. Test-retest reliability, assessed in a random subsample of 20 women, revealed an intraclass correlation coefficient (ICC) of 0.84 (95% confidence interval (CI): 0.78-0.90), indicating good temporal stability.

Construct validity

Model performance was satisfactory, with Kaiser-Meyer-Olkin (KMO) sampling adequacy=0.81. KMO values above 0.80 are generally interpreted as evidence of meritorious sampling adequacy, confirming that the dataset was appropriate for the extraction of latent components and that common variance was substantial enough to justify factor retention. Bartlett’s test of sphericity was significant (χ²=412.3, p<0.001), further supporting the presence of correlations suitable for dimensional reduction.

Exploratory factor analysis with principal component analysis and Varimax rotation supported a five-factor solution, explaining 68% of the total variance. All 24 items demonstrated primary loadings ≥0.40 on their intended domains, with minimal cross-loadings (<0.30). Detailed factor loadings are presented in Table 2. Internal consistency was high (Cronbach’s α=0.89 for the total scale; 0.78-0.86 for subscales). Test-retest reliability was good (ICC=0.84). Inter-domain correlations ranged from r=0.40 to r=0.52, indicating moderate associations without redundancy. Variance inflation factors (VIF) were <2.0 for all domains, excluding multicollinearity.

**Table 2:** Exploratory factor analysis of the SSEI-CS-24 (PCA with Varimax rotation) Each item demonstrated a loading ≥0.40 on its intended domain, supporting the construct validity of the five-factor model. Cross-loadings on other domains were minimal (<0.30) and not reported. Only primary loadings ≥0.40 are shown. The five domains correspond to sexual satisfaction (items 1–5), body image and self-esteem (items 6–10), anxiety and stress (items 11–15), interpersonal relationships and intimacy (items 16–20), and support and resources (items 21–24). PCA=principal component analysis. Varimax rotation applied. *Items were reverse-coded prior to analysis. Item wording is presented in abbreviated form for readability; full item text is provided in Appendix A.

Item	Item wording (abbreviated)	Factor loading
1	I am satisfied with my sexual life after cesarean delivery	0.74
2	My sexual desire is the same as before cesarean delivery	0.71
3	I need to adjust sexual positions because of the scar*	0.76
4	I feel relaxed and confident during sexual activity	0.68
5	The scar causes pain or discomfort during intercourse*	0.73
6	I feel comfortable with my body image despite the scar	0.81
7	The postoperative scar negatively affects my self-esteem*	0.79
8	I feel ashamed or embarrassed because of the scar*	0.72
9	I feel confident undressing in front of my partner	0.75
10	I am satisfied with the appearance of my scar	0.77
11	The scar has caused significant anxiety or stress*	0.70
12	I experienced depressive symptoms related to the scar*	0.74
13	I feel fear or worry because of the scar*	0.78
14	I feel calm and emotionally balanced despite the scar	0.72
15	The scar has caused sleep disturbances*	0.69
16	My partner has been supportive regarding my scar	0.73
17	The scar negatively affected my relationship*	0.75
18	I feel equally desired by my partner	0.70
19	I feel emotionally connected to my partner during sex	0.72
20	The scar reduced the frequency of sexual intercourse*	0.76
21	I received adequate emotional support after delivery	0.80
22	Psychological counseling after cesarean delivery would be useful	0.78
23	I have access to adequate recovery resources	0.74
24	I feel encouraged by family and friends	0.82

**Figure 2:**
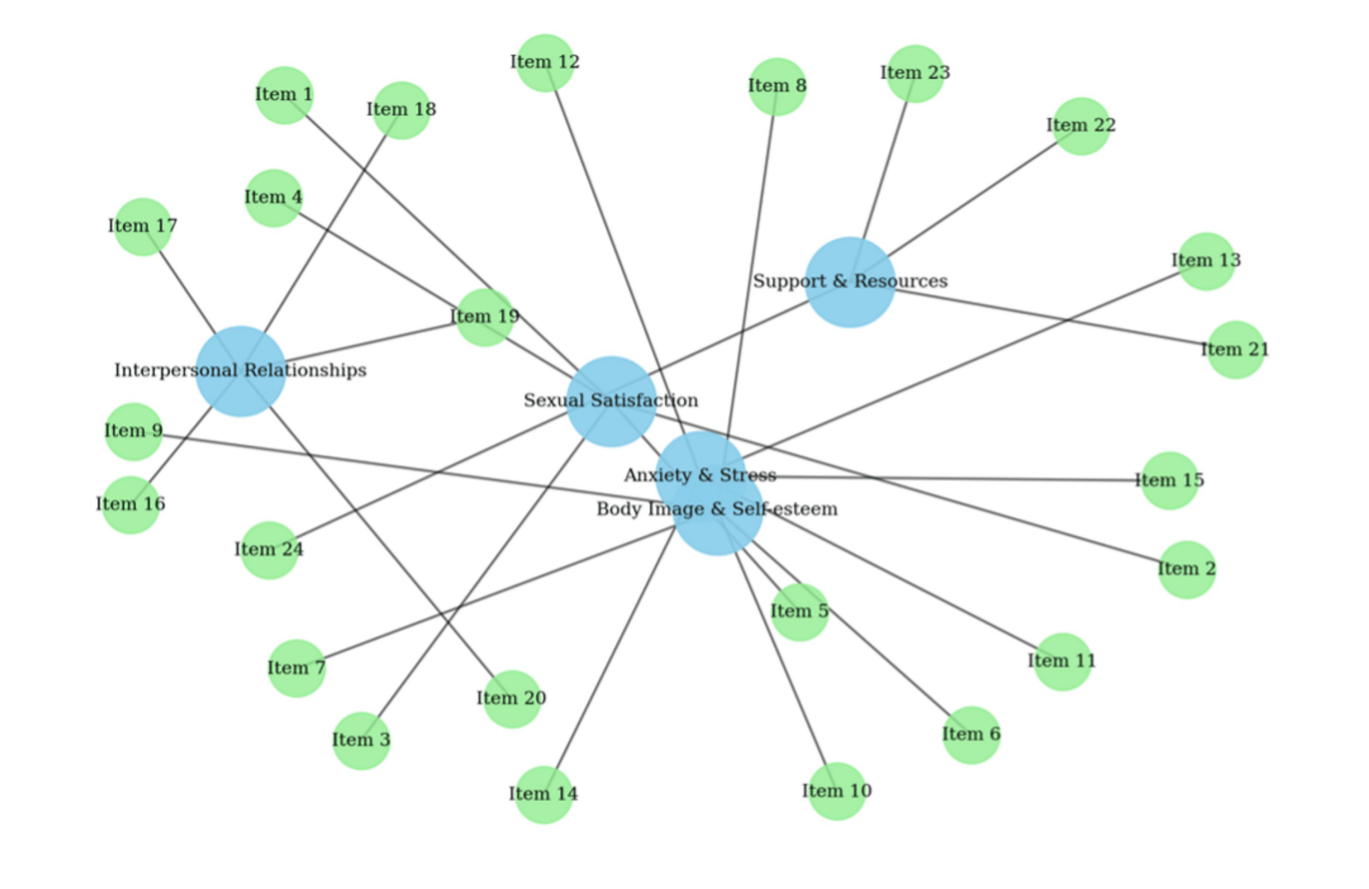
Factorial structure of the SSEI-CS-24 (Exploratory Factor Analysis) The diagram illustrates the five-factor solution identified through principal component analysis with Varimax rotation. Blue nodes represent the latent domains - sexual satisfaction (items 1-5), body image and self-esteem (items 6-10), anxiety and stress (items 11-15), interpersonal relationships and intimacy (items 16-20), and support and resources (items 21-24) - while green nodes represent the individual items. Each item demonstrated a primary loading ≥0.40 on its intended domain, confirming the hypothesized factorial structure of the instrument. SSEI-CS-24: Sexual Satisfaction and Emotional Impact after Cesarean Section Scale

**Figure 3:**
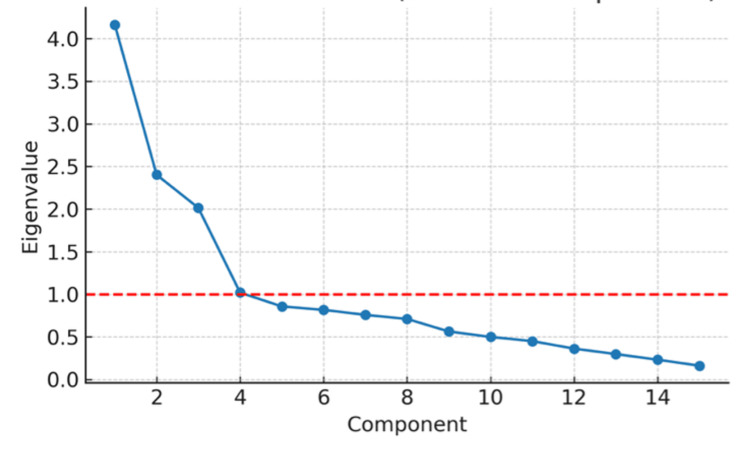
Scree Plot of Principal Component Analysis The scree plot displays the eigenvalues of the 24 items, showing a gradual decline in explained variance across components. Although the inflection point appears after the third component, five components had eigenvalues greater than 1.0 and were therefore retained based on both statistical criteria and the theoretical framework underlying the scale. This supports the five-factor structure identified through exploratory factor analysis.

The factorial structure is further illustrated in Figure 2, which shows the clustering of items across the five latent domains: sexual satisfaction, body image and self-esteem, anxiety and stress, interpersonal relationships and intimacy, and support and resources. The scree plot (Figure 3) confirmed the five-factor structure. Item-level CVI (I-CVI) ranged from 0.82 to 0.96, while the scale-level CVI (S-CVI/Ave) reached 0.91, exceeding the ≥0.80 threshold.

Given the minimal sample size relative to the number of items, the exploratory factor analysis should be interpreted with extreme caution. The study was conducted solely to explore preliminary item clustering and internal coherence rather than to establish a stable latent structure. The results do not constitute evidence of construct validity and are presented only to inform future scale refinement and adequately powered validation studies.

The variables corresponding to each latent domain - sexual satisfaction (items 1-5), body image and self-esteem (items 6-10), anxiety and stress (items 11-15), interpersonal relationships and intimacy (items 16-20), and support and resources (items 21-24) - as presented in Table 2, are fully detailed in the Appendixes A and B.

Although the scree plot showed a sharp decline after the third component, we retained a five-factor solution. This decision was based on both statistical and theoretical considerations: eigenvalues for factors four and five remained >1.0, the five-factor model explained 68% of variance, and it aligned with the a priori conceptual framework of the instrument. These findings should be interpreted as exploratory, given the limited sample size. Confirmatory factor analysis (CFA) was not feasible in this pilot study and will be required in larger validation cohorts.

Distribution of scores

**Figure 4:**
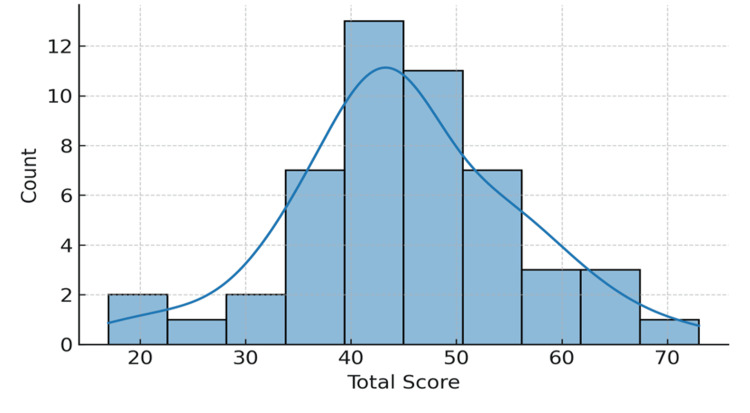
Distribution of the SSIE-CS-24 Total Score The histogram shows the distribution of total SSEI-CS-24 scores among the 50 patients. The distribution approximates a normal curve, with mean scores centered around the mid-range of the scale. This suggests that the instrument captures a balanced spread of responses without significant floor or ceiling effects, supporting its validity for clinical and research use. SSEI-CS-24: Sexual Satisfaction and Emotional Impact after Cesarean Section Scale

The total SSEI-CS-24 score ranged between 55 and 112, with a mean of 81.7±13.2. The histogram of the total score (Figure 4) approximated a normal curve, with no evidence of floor or ceiling effects (<15% of participants reached extreme values). This distribution supports the discriminatory capacity and validity of the scale for both clinical and research use. No participants reached the minimum or maximum possible scores, excluding the risk of floor or ceiling effects.

Histograms of domain and total scores were inspected for normality, skewness, and floor/ceiling effects. A distribution was deemed acceptable if <15% of participants reached the lowest or highest possible score.

Distributions were approximately normal with sufficient variability to ensure good discrimination among the respondents. The distribution confirms adequate variability of responses, ensuring good psychometric discrimination across items (Table 3).

**Table 3:** Descriptive statistics of SSEI-CS-24 All five SSEI-CS-24 subscales showed adequate dispersion, with means ranging between 14.7 and 18.2 and standard deviation (SD) values between 3.8 and 4.5. The observed score ranges (5–25) indicate appropriate variability without floor or ceiling effects, supporting the instrument’s discriminative capacity across domains. SD: Standard deviation.

Subscale	Mean	SD	Range (min–max)
Sexual Satisfaction	15.4	4.2	6-24
Body Image and Self-esteem	16.1	3.9	7-24
Anxiety and Stress	14.7	4.5	5-25
Interpersonal Relationships and Intimacy	17.3	3.8	8-25
Support and Resources	18.2	4.1	7-25

Correlations between domains

Pearson’s analysis revealed significant associations. Sexual Satisfaction correlated positively with Body Image (r=0.52, p<0.001) and Interpersonal Relationships and Intimacy (r=0.48, p<0.01). Anxiety and Stress correlated negatively with Body Image (r=-0.44, p<0.01) and Sexual Satisfaction (r=-0.41, p<0.05). Support and Resources correlated positively with the total score (r=0.40, p<0.01).

## Discussion

The present study reports the development and pilot validation of SSEI-CS-24, a novel instrument specifically designed to capture the multidimensional psychosocial consequences of cesarean section scars. The SSEI-CS-24 demonstrated promising psychometric properties in this pilot validation. However, its readiness for clinical application remains preliminary. Larger multicenter studies are needed to conduct CFA, assess responsiveness to clinical change, and validate the scale against established instruments such as the FSFI and EPDS.

To our knowledge, this is the first validated instrument that comprehensively integrates the sexual, psychological, and relational scar-related impact into a single scale. Existing tools such as FSFI and EPDS capture only partial aspects of postpartum well-being [18,19]. The FSFI is primarily designed to assess sexual function in general populations and does not address postpartum-specific issues such as scar-related discomfort, body image, or partner intimacy [18]. Conversely, the EPDS focuses exclusively on depressive symptoms, without considering sexual function, relational dynamics, or scar perception [19]. The SSEI-CS-24 fills this critical gap by simultaneously assessing sexual satisfaction, body image, emotional distress, partner intimacy, and social support domains that together provide a holistic picture of women’s adaptation after cesarean delivery.

Our findings confirmed that sexual satisfaction is strongly linked to body image and self-esteem, consistent with prior studies showing that postpartum body dissatisfaction is a predictor of impaired sexual functioning and depression [20,21]. Women with better body image adaptation reported significantly higher satisfaction scores, underlining the importance of psychological interventions aimed at improving self-perception after birth [22]. The Anxiety and Stress subscale displayed the greatest variability, reflecting the heterogeneous emotional burden experienced by women after cesarean delivery. Previous research has suggested that cesarean section is associated with a higher risk of postpartum depression and anxiety compared to vaginal birth, though findings remain inconclusive [7,22]. The SSEI-CS-24 allows clinicians to identify women at risk of psychological maladaptation and to tailor interventions such as counseling or support groups.

Consistent with the literature, social support emerged as a significant protective factor, positively correlating with higher overall adaptation scores [23]. Interpersonal relationships and perceived partner support are known mediators of sexual function recovery, emotional stability, and long-term well-being [24,25]. The association with interpersonal relationships highlights that partner intimacy and relational support are essential facilitators of sexual recovery. By explicitly including this dimension, the SSEI-CS-24 increases its clinical value, guiding interventions beyond the individual to also target family and relational dynamics.

The scale’s ability to discriminate between women with low, moderate, and high adaptation suggests its utility both as a screening tool in routine postpartum care and as an outcome measure in research trials evaluating interventions such as surgical techniques, wound-healing agents, or psychosocial support programs. For instance, adjunctive therapies such as platelet-rich plasma (PRP) or lactic acid-based agents for improved wound healing may also have indirect benefits on sexual and emotional recovery, as suggested by previous studies. These potential associations could be further explored by correlating scar quality with psychosocial adaptation in future trials [26].

A major strength of this pilot validation lies in the methodological rigor, including expert-driven item development, comprehensive psychometric testing, and alignment with international guidelines for scale validation [13,14]. Nevertheless, this study has limitations. The sample size was modest (n=50), consistent with pilot study recommendations but insufficient for CFA [27]. Moreover, the scale was tested only at 40 days postpartum, and longitudinal validation is needed to capture the dynamic trajectory of recovery. Cross-cultural validation will also be essential to ensure generalizability across diverse populations.

While the scree plot alone could suggest a more parsimonious three-factor model, we favored the five-factor solution due to its alignment with the theoretical domains identified during instrument development and its satisfactory psychometric performance.

Although the scree plot suggests a more pronounced inflection after the third component and the eigenvalue of the fifth component was slightly below 1.0, factor retention was not based solely on the Kaiser criterion. Given the exploratory nature of this pilot study, factor retention was guided by a combination of statistical indicators and theoretical considerations established during instrument development. The five-factor solution demonstrated coherent item clustering, meaningful clinical interpretability, acceptable factor loadings, and explained a substantial proportion of total variance (68%). Therefore, the five-factor model was retained as a preliminary structure to be formally tested in future confirmatory factor analysis using larger, independent samples.

Body image and sexual satisfaction

Our results confirmed a significant association between body image and sexual satisfaction, underscoring the psychological dimension of postpartum sexual health. Women who reported greater body image acceptance demonstrated higher levels of sexual satisfaction, a finding consistent with prior research highlighting that postpartum body dissatisfaction is a strong predictor of impaired sexual function and depressive symptoms. Studies published in high-impact journals further corroborate this link. For instance, studies demonstrated that negative body perception after childbirth was independently associated with decreased sexual activity and dyspareunia [4,22]. Similarly, an analysis in the *Journal of Obstetrics and Gynaecology Research* reported that body image concerns mediated the relationship between mode of delivery and female sexual function, particularly in women undergoing cesarean section [28]. In addition, a recent multicenter cohort from the *Journal of Psychosomatic Obstetrics and Gynecology* highlighted that poor body image and scar dissatisfaction were among the strongest predictors of persistent declines in sexual satisfaction at one year postpartum [29].

These findings reinforce the clinical relevance of integrating body image assessment into postpartum care. The SSEI-CS-24 uniquely captures this dimension, providing healthcare professionals with an evidence-based tool to recognize women who may benefit from targeted psychological support aimed at improving self-esteem, promoting positive body image, and thereby enhancing sexual well-being.

Clinical implications

The SSEI-CS-24 offers clinicians a structured approach to exploring concerns related to intimacy, emotional balance, and partner dynamics following cesarean birth. When incorporated into postpartum consultations, the scale may help identify individuals who could benefit from targeted counseling or supportive interventions. In research settings, its multidimensional structure makes it a suitable tool for evaluating the impact of surgical techniques, scar management methods, or psychological support programs on women’s recovery trajectories.

Limitations

A key limitation is the modest sample size (n=50) relative to the 24-item scale, which constrains the stability and generalizability of the exploratory factor solution. While commonly cited heuristics suggest larger samples (e.g., five to 10 participants per item), we intended to conduct an initial exploratory assessment to inform subsequent validation. Future multicenter studies with substantially larger samples will be required to perform CFA, assess measurement invariance, and confirm the factor structure before routine clinical use.

The cut-off values presented are exploratory and provisional. Clinically meaningful thresholds will require empirical validation against external criteria in larger, multicenter cohorts.

Another limitation that should be acknowledged is that no categorical thresholds were defined in this pilot study, as clinically meaningful cut-offs will need to be determined in future studies by correlating SSEI-CS-24 scores with established instruments such as EPDS or FSFI. This comparison will be essential in future validation studies to confirm the convergent and discriminant validity of the SSEI-CS-24.

Another important limitation is that all participants were recruited from a single center in Romania. While this allowed for methodological consistency, it restricts the cultural generalizability of our findings. Cross-cultural validation in larger and more heterogeneous samples will be essential to confirm the scale’s applicability in different sociocultural contexts.

The timing of assessment at 40 days may not fully capture the resumption of sexual activity, which often occurs between six and eight weeks postpartum. However, sexual satisfaction, scar-related impact, and emotional adaptation may continue to evolve over subsequent months. Longitudinal validation at three, six, and 12 months will be essential to fully establish the instrument’s validity across the postpartum trajectory [30].

Future research should focus on confirmatory validation with larger and more heterogeneous samples, cross-cultural adaptation to account for sociocultural influences on body image and sexual functioning, and longitudinal designs tracking recovery up to one year postpartum. Integration with clinical trials assessing interventions such as scar management, counseling, and physical rehabilitation will also help determine the instrument’s responsiveness to change. In subsequent phases, external validity will be examined by correlating SSEI-CS-24 with the FSFI and EPDS, and by subgroup analyses according to the type of cesarean section (elective vs. emergency).

## Conclusions

In summary, the SSEI-CS-24 is the first validated instrument specifically developed to comprehensively assess the sexual, psychological, and relational impact of cesarean section scars. The scale demonstrated strong psychometric properties, including excellent internal consistency, satisfactory reliability, and a coherent factorial structure consistent with theoretical domains. By integrating multiple dimensions of postpartum adaptation into a single tool, the SSEI-CS-24 provides clinicians and researchers with a reliable means of identifying women at risk of maladaptation, monitoring recovery, and evaluating the effectiveness of clinical or psychosocial interventions. Future larger-scale and cross-cultural validation is warranted before widespread clinical adoption. Given its pilot nature, the SSEI-CS-24 should currently be regarded as an exploratory instrument. Future work will be essential to confirm its psychometric robustness and clinical applicability.

## Appendices

Appendix A

Sexual Satisfaction and Emotional Impact after Cesarean Section Scale (SSEI-CS-24, English version)

This scale is intended to evaluate the impact of cesarean section scars on sexual life and psychological well-being at 40 days postpartum.

Each statement should be rated on a five-point Likert scale:

1=Strongly disagree 2=Disagree 3=Neutral 4=Agree 5=Strongly agree

A. Sexual Satisfaction

1. I am satisfied with my sexual life after cesarean delivery.

2. My sexual desire is the same as before the cesarean delivery.

3. I need to change or adjust sexual positions because of the scar (reverse coded).

4. I feel relaxed and confident during sexual activity.

5. The scar causes pain or discomfort during intercourse (reverse coded).

B. Body Image and Self-Esteem

6. I feel comfortable with my body image despite the scar.

7. The postoperative scar negatively affects my self-esteem (reverse coded).

8. I feel ashamed or embarrassed because of the scar (reverse coded).

9. I feel confident undressing in front of my partner despite the scar.

10. I am satisfied with the appearance of my postoperative scar.

C. Anxiety and Stress

11. The postoperative scar has caused significant anxiety or stress (reverse coded).

12. I have experienced depressive symptoms related to the postoperative scar (reverse coded).

13. I feel fear or worry because of the scar (reverse coded).

14. I feel calm and balanced despite the presence of the postoperative scar.

15. The scar has caused sleep disturbances or insomnia (reverse coded).

D. Interpersonal Relationships and Intimacy

16. My partner has been supportive and understanding regarding my scar.

17. The postoperative scar has negatively affected my relationship with my partner (reverse coded).

18. I feel equally desired by my partner despite the scar.

19. I feel emotionally connected to my partner during sexual activity.

20. The scar has reduced the frequency of sexual intercourse after cesarean delivery (reverse coded).

E. Support and Resources

21. I have received adequate emotional support after the cesarean delivery.

22. I believe that psychological counseling specifically for women after cesarean delivery would be useful.

23. I have access to adequate resources and information regarding postoperative recovery.

24. I feel encouraged by the support received from family and friends.

Scoring and Interpretation

Total Score=sum of all items (range: 24-120). Higher scores indicate greater sexual satisfaction, better self-esteem, lower anxiety/stress, stronger interpersonal relationships, and better perceived support.

Suggested interpretation

97-120: Very high satisfaction and psychological adaptation 73-96: Moderate satisfaction and adaptation 49-72: Low satisfaction and adaptation <48: Very low satisfaction and adaptation

The proposed cut-off values (very low, low, moderate, high) are provisional and intended only for illustrative use in this pilot phase. They do not represent clinically validated thresholds and should be redefined in future validation studies through correlation with established instruments (e.g., FSFI, EPDS).

Appendix B

Sexual Satisfaction and Emotional Impact after Cesarean Section Scale (SSEI-CS-24, Romanian original version)

Această scală este destinată evaluării impactului cicatricei postoperatorii asupra vieții sexuale și stării psihologice la 40 de zile după nașterea prin operație cezariană.

Pentru fiecare afirmație, vă rugăm să indicați gradul în care sunteți de acord sau nu sunteți de acord, pe o scală de la 1 la 5:

1 = Total dezacord 2 = Dezacord 3 = Neutru 4 = Acord 5 = Total acord

A. Satisfacția sexuală

1. Sunt mulțumită de viața mea sexuală după operația cezariană.

2. Am aceeași dorință sexuală ca înainte de operația cezariană.

3. Am nevoie de schimbări sau ajustări ale pozițiilor sexuale din cauza cicatricei.

4. Mă simt relaxată și încrezătoare în timpul activităților sexuale.

5. Cicatricea provoacă durere sau disconfort în timpul actului sexual.

B. Stima de sine și imaginea corporală

6. Mă simt confortabilă cu imaginea corpului meu, în ciuda cicatricei.

7. Cicatricea postoperatorie îmi afectează stima de sine.

8. Mă simt rușinată sau jenată din cauza cicatricei.

9. Mă simt încrezătoare să mă dezbrac în fața partenerului meu, în ciuda cicatricei.

10. Sunt mulțumită de aspectul cicatricei postoperatorii.

C. Anxietate și stres

11. Cicatricea postoperatorie a cauzat anxietate sau stres semnificativ.

12. Am experimentat simptome de depresie legate de cicatricea postoperatorie.

13. Simt teamă sau îngrijorare legată de cicatricea postoperatorie.

14. Mă simt calmă și echilibrată în ciuda cicatricei postoperatorii.

15. Cicatricea îmi cauzează probleme de somn sau insomnie.

D. Relații interpersonale și intimitate

16. Partenerul meu a fost susținător și înțelegător în legătură cu cicatricea mea.

17. Cicatricea postoperatorie a influențat negativ relația mea de cuplu.

18. Mă simt la fel de dorită de partenerul meu, în ciuda prezenței cicatricei.

19. Mă simt conectată emoțional cu partenerul meu în timpul actului sexual.

20. Cicatricea a redus frecvența actelor sexuale după operația cezariană.

E. Suport și resurse

21. Am beneficiat de suport emoțional adecvat după operația cezariană.

22. Consider că consilierea psihologică specifică pentru femeile care au trecut printr-o operație cezariană ar fi utilă.

23. Am acces la resurse și informații utile privind recuperarea postoperatorie.

24. Mă simt încurajată de sprijinul primit de la familie și prieteni.

Scor total și interpretare

Scor total (suma punctelor): _____ / 120 97-120: Satisfacție și adaptare psihologică foarte mare 73-96: Satisfacție și adaptare psihologică moderată 49-72: Satisfacție și adaptare psihologică scăzută <48: Satisfacție și adaptare psihologică foarte scăzută

* Valorile prag propuse (foarte scăzut, scăzut, moderat, ridicat) sunt provizorii și destinate exclusiv unei utilizări ilustrative în această fază pilot. Ele nu reprezintă praguri validate clinic și ar trebui redefinite în studii viitoare de validare, prin corelare cu instrumente consacrate (de exemplu, FSFI, EPDS).
